# deTELpy: Python package for high-throughput detection of amino acid substitutions in mass spectrometry datasets

**DOI:** 10.1093/bioinformatics/btae424

**Published:** 2024-06-28

**Authors:** Cedric Landerer, Maxim Scheremetjew, HongKee Moon, Lena Hersemann, Agnes Toth-Petroczy

**Affiliations:** Max Planck Institute of Molecular Cell Biology and Genetics, 01307 Dresden, Germany; Center for Systems Biology Dresden, 01307 Dresden, Germany; Max Planck Institute of Molecular Cell Biology and Genetics, 01307 Dresden, Germany; Center for Systems Biology Dresden, 01307 Dresden, Germany; Max Planck Institute of Molecular Cell Biology and Genetics, 01307 Dresden, Germany; Center for Systems Biology Dresden, 01307 Dresden, Germany; Max Planck Institute of Molecular Cell Biology and Genetics, 01307 Dresden, Germany; Center for Systems Biology Dresden, 01307 Dresden, Germany; Max Planck Institute of Molecular Cell Biology and Genetics, 01307 Dresden, Germany; Center for Systems Biology Dresden, 01307 Dresden, Germany; Cluster of Excellence Physics of Life, TU Dresden, 01062 Dresden, Germany

## Abstract

**Motivation:**

Errors in the processing of genetic information during protein synthesis can lead to phenotypic mutations, such as amino acid substitutions, e.g. by transcription or translation errors. While genetic mutations can be readily identified using DNA sequencing, and mutations due to transcription errors by RNA sequencing, translation errors can only be identified proteome-wide using mass spectrometry.

**Results:**

Here, we provide a Python package implementation of a high-throughput pipeline to detect amino acid substitutions in mass spectrometry datasets. Our tools enable users to process hundreds of mass spectrometry datasets in batch mode to detect amino acid substitutions and calculate codon-specific and site-specific translation error rates. deTELpy will facilitate the systematic understanding of amino acid misincorporation rates (translation error rates), and the inference of error models across organisms and under stress conditions, such as drug treatment or disease conditions.

**Availability and implementation:**

deTELpy is implemented in Python 3 and is freely available with detailed documentation and practical examples at https://git.mpi-cbg.de/tothpetroczylab/detelpy and https://pypi.org/project/deTELpy/ and can be easily installed via pip install deTELpy.

## 1 Introduction

Proteins owe their name to the fact that they are the primary functional molecules of a cell (Vickery 1950). Despite the importance of proteins, their production is an error-prone process with transcription and translation error rates far exceeding genetic mutation rates. Transcriptome-wide transcription error rates can be measured using high fidelity RNA-sequencing techniques resulting in estimates of 10^−6^–10^−5^ nucleotide misincorporation per codon in various organisms (Gout *et al.* 2017, Li and Lynch 2020). However, translation error rates are often measured only for individual proteins and specific residue sites using e.g. fluorescent reporter assays. These assays revealed a large variability in translation fidelity across error types, such as amino acid misincorporations, frameshifts and stop codon readthrough, as well as variation across organisms reviewed in (Romero Romero *et al.* 2022). Reporter assay of individual codon positions measured a wide range of error rates from 10^−6^–10^−3^ (Edelmann and Gallant 1977, Parker *et al.* 1983, Khazaie *et al.* 1984, Toth *et al.* 1988, Kramer and Farabaugh 2007). Only recently have been proteome-wide studies of translation errors, specifically amino acid misincorporations, possible using high coverage mass spectrometry proteomics (Mordret *et al.* 2019) and high-throughput mass spectrometry data processing (Landerer *et al.* 2024). These studies revealed an average error rate ranging from ∼10^−5^ to ∼10^−3^ amino acid misincorporation per codon in *Escherichia coli and Saccharomyces cerevisiae*. Overall, translation errors are frequent and on average ∼20% of proteins synthesized in the cell are expected to harbor at least one amino acid misincorporations (Landerer *et al.* 2024). The above-mentioned studies used different ways of quantifying amino acid misincorporations. Mordret *et al.* defined “site specific error rates” based on mass spectrometry peak intensities: they calculated the ratio of MS1 peak intensities of peptides with amino-acid misincorporations and the peptides without (error-free peptides). Landerer *et al.* defined “codon aggregated error rates” based on peptide counts: the number of times codon i was observed to be mistranslated divided by the total number of times codon i was observed in any peptide. Both studies used custom pipelines and code to identify peptides with amino acid misincorporations, since there is no standard data processing workflow and software that can readily do that.

Here, we present deTELpy, a python package consisting of three modules, which allow the identification of amino acid misincorporations and the study of translation error rates from proteome-wide mass-spectrometry data. deTELpy incorporates both previously described workflows and algorithms (Mordret *et al.* 2019, Landerer *et al.* 2024) and streamlines data processing. The first module is revealing Translation Error Landscape (rTEL) which is an easy-to-use interface to the *open search* module (Yu *et al.* 2020b) of MSFragger (Kong *et al.* 2017) with predefined settings which can be accessed and modified for a specific application in rTEL, and allows for the detection of mass-shifts. The second module is empirical Translation Error Landscape (eTEL) which filters the rTEL output, removing all peptides with mass-shifts that cannot be uniquely assigned as amino acid replacements. The third module is a mechanistic Translation Error Landscape (mTEL) model, that can be fitted to the observed data from eTEL to extrapolate to unobserved translation errors. mTEL’s only additional input data is tRNA abundance, that is necessary for modeling competition for tRNA arrival, which contributes to tRNA misincorporation (Landerer *et al.* 2024). Overall, deTELpy is easily applicable to a wide range of species. For example, the mTEL model has been already applied by our group to test the effects of tRNA abundance or replacement (Landerer *et al.* 2024).

So far, deTEL was successfully used to detect translation errors in ∼100 *E.coli* and *S.cerevisiae* mass-spectrometry datasets by (Landerer *et al.* 2024). Mistranslation and tRNA dynamics were found to play important roles in stress (Mohler and Ibba 2017) and diseases (Kirchner and Ignatova 2015), such as cancer (Goodarzi *et al.* 2016, Pataskar *et al.* 2022). With deTELpy, it is now possible to efficiently analyze translation accuracy in multiple conditions as well as across different species.

## 2 Materials and methods

**Figure 1.**
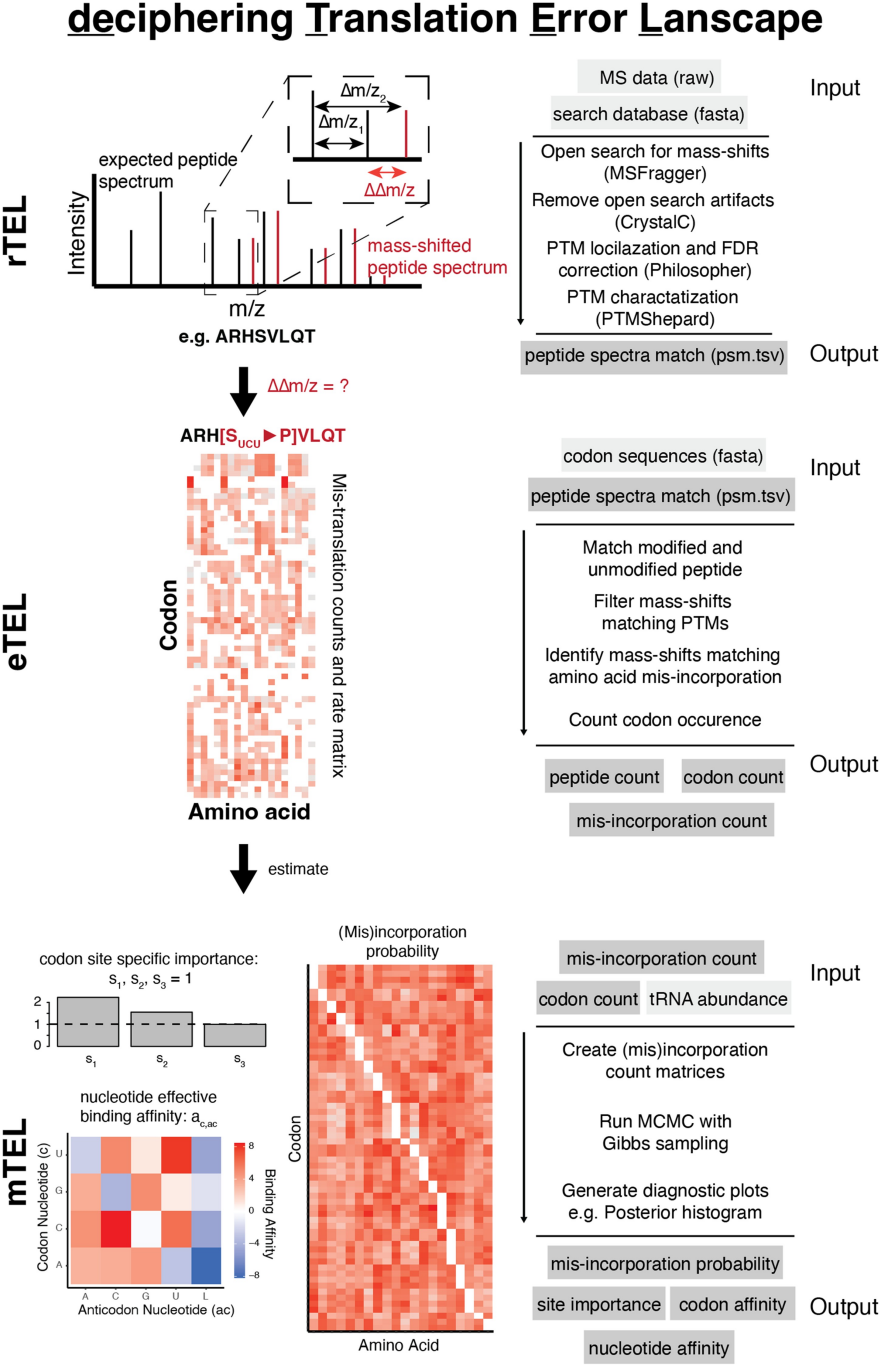
The modular workflow of deTELpy allows customized workflows for the detection of amino acid substitutions. deTELpy has been developed with high performance clusters in mind and simplifies the detection of amino acid substitutions across many datasets. The rTEL module is a wrapper to the FragPipe open search using MSFragger with predefined parameters. Open search parameters can be adjusted if needed. The eTEL module filters the output of rTEL and extracts mass shifted peptides matching uniquely assignable amino acid substitutions. It utilizes the *unimod* database to only retain mass shifts that cannot be attributed to other post translational modifications. eTEL can be run independently with user provided input files from MSFragger or with the output files generated by rTEL. mTEL is a translation model, accounting for tRNA abundance and codon/anticodon binding and is fitted to the eTEL output. mTEL outputs fitted codon/anticodon binding interactions, and codon/amino acid (mis)incorporation probabilities. Additional input files, not generated by the pipeline, are highlighted in light gray.

deTELpy (deciphering Translation Error Landscape) contains three modules, rTEL, eTEL, and mTEL, and is implemented in Python (Fig. 1). deTELpy is designed for easy usage even for users without excessive computational or mass-spectrometry experience and provides the functionality to be also easily run on HPC systems. Additionally, we included an optional GUI for all modes (rTEL, eTEL, mTEL) for further ease of use. The rTEL function allows to process Thermo-Fisher “*.raw”* files and detect mass-shifts between observation and reference. The obtained mass-shifts are used by eTEL to identify amino acid misincorporations and remove mass-shifts best explained by post translational modifications (PTM). The observed amino acid misincorporations can then be used to fit the mTEL model in order to extrapolate to unobserved amino acid misincorporations. mTEL considers competition of tRNAs for arrival at the ribosome, based on their abundance, and the binding affinity between codon and anticodon nucleotides, fitted based on the empirical translation error landscape.

### 2.1 rTEL function

The *revealing* Translation Error Landscape (rTEL) function allows to identify mass-shifts of potential amino acid misincorporation. The function requires Thermo-Fisher “*.raw*” files and a “*fasta”* file of protein sequences to search against. rTEL is an interface to the open search workflow (Yu *et al.* 2020b) defined in FragPipe. rTEL uses predefined configurations that can be generated with “*—generate-config*” and adjusted and used in analysis if needed. Due to the easily usable Python interface, it enables parallel processing of multiple datasets on HPC systems. The open search allows for the detection of modified peptides based on mass-shifts between expected and observed peptide spectra, like it is the case if an amino acid is modified or replaced. The open search is performed using MSFragger (Kong *et al.* 2017) and the results are refined using Crystal-C (Chang *et al.* 2020) and False Discovery Rate corrected using philosopher (da Veiga Leprevost *et al.* 2020).

IonQuant can be used for label free peptide quantification (via the “*—ionquant*” option) which can be used by eTEL to calculate position specific misincorporation probabilities (Yu *et al.* 2020a, Yu *et al.* 2021). The rTEL function outputs a peptide-spectrum match (“*psm*”) file which is passed on to the eTEL function for filtering and detection of amino acid misincorporation.

### 2.2 eTEL function

The *empirical* Translation Error Landscape (eTEL) function uses the output “*psm”* file from either the rTEL function or the fragpipe open search workflow as input. eTEL filters the observed mass-shifts for translation errors, which can be uniquely assigned to an individual position in a peptide. Examples of the peptide identification counts and the filtering process are shown in Supplementary Fig. S1 and Supplementary Table S1.

In addition, eTEL provides translation error rate estimates based on two algorithms. By default, it computes the error detection rate from the codon counts observed in modified and unmodified peptides as described in Landerer *et al.* (2024). It can also calculate site-specific error rates based on peptide intensities as described in Mordret *et al.* (2019), if enabled in the rTEL function (*—ionquant*). eTEL requires a coding sequence version of the reference proteome (e.g. obtained from Ensembl) used for the open search performed by rTEL. eTEL only considers mass-shifts that are (i) observed in peptides which were also detected without mass-shifts (a.k.a without substitutions), (ii) can be uniquely assigned to a position, (iii) cannot be explained by other types of post-translational modifications. eTEL outputs three files for each *“psm*” file; (i) peptide counts containing the number of peptides identified for each protein, with and without error, as well as the error rate of a protein; (ii) codon counts containing the observed codons and their error rate; (iii) the substitution file, containing the mistranslated codon, it’s location and the observed misincorporated amino acid.

### 2.3 mTEL function

The *mechanistic* Translation Error Landscape (mTEL) function is a probabilistic model, explicitly modeling the competition between tRNAs for arrival, and the codon/anticodon binding. The mTEL algorithm is described in detail in (Landerer *et al.* 2024). mTEL requires the codon count and the observed substitution from eTEL as well as tRNA abundance data as additional input in “*.tsv*.” Example files for *E.coli* and *S.cerevisiae* are provided as part of deTELpy. While arrival rates are calculated from provided tRNA abundance data, codon/anticodon binding parameters are fitted to the observed mis-incorporations detected by the eTEL function. Codon/anticodon binding parameters are a linear combination of individual nucleotide binding parameters and site-specific importance parameters for each codon position, minimizing the actual number of inferred parameters. mTEL outputs the estimated parameters (nucleotide binding affinity and site-specific importance) as well as codon binding affinity and amino acid misincorporation probability calculated form the fitted parameters.

## 3 Usage

To demonstrate the usage of deTELpy, we provide an example workflow including input datafiles: *“.raw”* files obtained from PRIDE, tRNA abundance data and database *“fasta”* file. We use mass spectrometry data from Bilus *et al.* who studied valine and norvaline misincorporations due to an error-prone isoleucyl-tRNA-synthetase (Bilus *et al.* 2019). Detailed description of the workflow and executable commands can be found on our GitLab https://git.mpi-cbg.de/tothpetroczylab/detelpy/-/releases/v0.1.13, on Zenodo https://doi.org/10.5281/zenodo.11257967 and at https://pypi.org/project/deTELpy/.

## 4 Conclusions

Phenotypic mutations, e.g. amino acid misincorporations as a result of transcription or translation errors contribute to protein evolution (Romero Romero *et al.* 2022). High-throughput identification of these individually rare events is challenging. Mass spectrometry proteomics allows for the detection and identification of peptides matching measured spectra to theoretical peptide spectra generated from a sequence database (Cox and Mann 2011). Many spectra, however, are not matched to any theoretical peptides, due to e.g. PTMs which alter the peptides' mass. Various approaches have been developed to account for such mass-shifts due to PTMs (Tang *et al.* 2005, Chick *et al.* 2015, Yu *et al.* 2020b). Treating amino acid misincorporations as post translational modifications (Mordret *et al.* 2019) allows deTELpy to identify amino acid misincorporations proteome-wide. Using deTELpy, it is now possible to explore the large variability in translation fidelity between many species indicated by reporter assays (Romero Romero *et al.* 2022), as well as across conditions, such as stress (Mohler and Ibba 2017) or diseases (Kirchner and Ignatova 2015), e.g. in cancer (Goodarzi *et al.* 2016, Pataskar *et al.* 2022).

## Supplementary Material

btae424_Supplementary_Data

## Data Availability

Test datasets are available at https://doi.org/10.17617/3.N58KMF.
