# Supplementary material for: deTELpy: Python package for high-throughput detection of amino acid substitutions in mass spectrometry datasets
Source: Bioinformatics. 2024 Jun 28;40(7):btae424. doi: 10.1093/bioinformatics/btae424 (PMC11236091; doi:10.1093/bioinformatics/btae424)
Supplement: btae424_Supplementary_Data [file btae424_supplementary_data.pdf]

Supplementary Information

for

deTELpy: Python package for high-throughput detection of amino acid substitutions in mass spectrometry datasets

Cedric Landerer[1,2], Maxim Scheremetjew[1,2], HongKee Moon[1,2], Lena Hersemann[1,2], Agnes Toth-Petroczy[1,2,3]

[1]Max Planck Institute of Molecular Cell Biology and Genetics, 01307 Dresden, Germany

[2]Center for Systems Biology Dresden, 01307 Dresden, Germany

[3]Cluster of Excellence Physics of Life, TU Dresden, 01062 Dresden, Germany

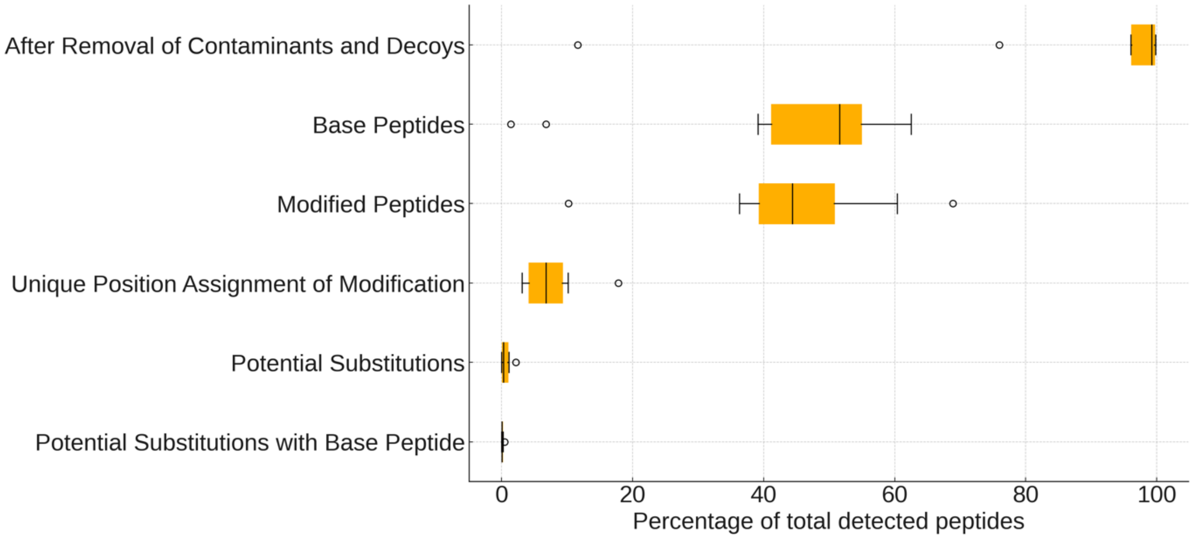

Supplementary Figure 1. Filtering and assignment of peptides with substitutions. Box plots represent the distribution of the percentages of peptides at each filtering step. Peptide counts were normalized to the total number of peptides observed for each dataset (N = 10). The black lines indicate the means across datasets and whiskers indicate standard deviations.

Supplementary Table 1. Representative dataset from PRIDE database (PXD ID - PRIDE data identifier) and their total number of identified peptides as well as the number of peptides after each fileting step in the rTEL pipeline. See also Supplementary Figure 1 for summary.

PXD ID	Total Peptides	After Removal of Contaminants and Decoys	Base Peptides	Modified Peptides	Unique Position Assignment of Modification	Potential Substitutions	Potential Substitutions with Base Peptide
**PXD008093**	20744	2405	292	2112	650	190	96
**PXD018291**	3728	2831	252	2568	148	0	0
**PXD005642**	296800	284984	161912	122190	12707	166	103
**PXD027167**	300195	299167	117384	181230	30529	2638	253
**PXD033577**	174230	168276	95761	71791	7201	665	69
**PXD024782**	324772	323738	167291	154922	31254	3670	133
**PXD022526**	133965	132614	78930	51794	10827	111	67
**PXD017618**	120170	119943	57073	62201	21438	234	163
**PXD009463**	472425	469717	244174	224577	32746	770	418
**PXD007087**	112021	111613	70025	40634	7469	2425	223
